# Gender differences in the association between childhood physical and sexual abuse, social support and psychosis

**DOI:** 10.1007/s00127-015-1058-6

**Published:** 2015-04-18

**Authors:** Charlotte Gayer-Anderson, Helen L. Fisher, Paul Fearon, Gerard Hutchinson, Kevin Morgan, Paola Dazzan, Jane Boydell, Gillian A. Doody, Peter B. Jones, Robin M. Murray, Thomas K. Craig, Craig Morgan

**Affiliations:** Centre for Epidemiology and Public Health, Health Service and Population Research Department, NIHR Biomedical Research Centre, Institute of Psychiatry, Psychology and Neuroscience, King’s College London, London, UK; MRC Social, Genetic and Developmental Psychiatry Centre, Institute of Psychiatry, Psychology and Neuroscience, King’s College London, London, UK; Department of Psychiatry, Trinity College Dublin, St Patrick’s University Hospital, Dublin, Ireland; Psychiatry Unit, University of the West Indies, Trinidad, Trinidad and Tobago; Department of Psychology, University of Westminster, London, UK; Psychosis Studies Department, NIHR Biomedical Research Centre, Institute of Psychiatry, Psychology and Neuroscience, King’s College, London, UK; Division of Psychiatry and Applied Psychology, University of Nottingham, Nottingham, UK; Department of Psychiatry, University of Cambridge, Cambridge, UK

**Keywords:** Onset, Psychosis, Childhood adversity, Social support, Social networks

## Abstract

**Purpose:**

Childhood adversity (variously defined) is a robust risk factor for psychosis, yet the mitigating effects of social support in adulthood have not yet been explored. This study aimed to investigate the relationships between childhood sexual and physical abuse and adult psychosis, and gender differences in levels of perceived social support.

**Methods:**

A sample of 202 individuals presenting for the first time to mental health services with psychosis and 266 population-based controls from south-east London and Nottingham, UK, was utilised. The Childhood Experience of Care and Abuse Questionnaire was used to elicit retrospective reports of exposure to childhood adversity, and the Significant Others Questionnaire was completed to collect information on the current size of social networks and perceptions of emotional and practical support.

**Results:**

There was evidence of an interaction between severe physical abuse and levels of support (namely, number of significant others; likelihood ratio test *χ*^2^ = 3.90, *p* = 0.048). When stratified by gender, there were no clear associations between childhood physical or sexual abuse, current social support and odds of psychosis in men. In contrast, for women, the highest odds of psychosis were generally found in those who reported severe abuse and low levels of social support in adulthood. However, tests for interaction by gender did not reach conventional levels of statistical significance.

**Conclusions:**

These findings highlight the importance of investigating the potential benefits of social support as a buffer against the development of adult psychosis amongst those, particularly women, with a history of early life stress.

**Electronic supplementary material:**

The online version of this article (doi:10.1007/s00127-015-1058-6) contains supplementary material, which is available to authorized users.

## Introduction

There is now strong evidence of increased risk of psychosis in those who have been exposed to severe adverse events in childhood, such as physical abuse, sexual abuse, psychological abuse, and bullying (see [1] for a meta-analysis of existing studies). The relationship between different types of maltreatment and psychosis, and whether the relationship varies by gender, has not been thoroughly explored. Preliminary evidence suggests that gender differences in the relationships between abuse and psychosis exist; that is, risk of psychosis following childhood sexual abuse appears to be higher in women compared with men [2–4]. Moreover, in our analyses of data from the Aetiology and Ethnicity in Schizophrenia and Other Psychoses (AESOP) study, we found that severe physical and sexual childhood abuse were associated with psychosis onset only in women [5].

However, not everyone who experiences trauma in childhood will go on to develop psychosis in adulthood. One moderating factor which has not been considered is the quality and quantity of perceived and received social support, which when lacking has been found to be robustly associated with other mental disorders such as depression [6, 7]. There are three broad dimensions of social support [8]: (a) social networks (e.g. number of contacts or frequency of contact); (b) perceived social support; and (c) enacted support, i.e. practical and emotional aid in the face of severe stress or daily hassles.

In particular, perceived support, the subjective belief that others are available to provide emotional and practical aid, has been shown to influence how individuals cope with stressful situations [9], possibly by way of shaping cognitive appraisals of stressful events [10]. In addition, individuals with a smaller social network have been shown to be more vulnerable to common mental disorders [7] and have a more severe course of depression [11]. There also appear to be discrepancies between men and women in the size of social networks and the use of social support resources, and this has been identified as the most robust difference in the way men and women cope with stress [12]. Women are more likely to utilise their social support systems in times of stress [13], and perceive support to be more adequate [14]. Despite more global support reported by women, Kendler and colleagues [15] found in a longitudinal twin study that a lack of social support was associated with increased risk of depression in women, but not in men.

In relation to psychosis, increasing evidence points towards reduced networks and lower perceived support preceding the onset of the disorder (see [16] for a review). In spite of this, there is a paucity of research on whether perceived social support or size of network in adulthood modifies the impact of childhood adversity on risk of psychosis. As childhood physical and sexual abuse are known to increase risk for adult psychosis, and social support in the form of perceived availability and size of network appears to promote resilience, the aim of this study was to investigate the relationship between these three variables. Extending our previous analyses of AESOP data, we hypothesised that there would be evidence of a three-way interaction such that, in women, larger social networks and greater perceived emotional and practical support would reduce the odds of having a psychotic disorder in those who had experienced severe early abuse.

## Method

### Sample

Data were collected as part of the Aetiology and Ethnicity in Schizophrenia and Other Psychoses (AESOP) study, a multi-centre population-based incidence and case–control study of first episode psychosis. Cases were included in the study if they were between 16 and 64 years old, lived within specific catchment areas in south-east London and Nottingham, UK, had a first episode of affective or non-affective psychosis (International Classification of Diseases (ICD-10 codes F20–F29 and F30–F33) [17] between 1997 and 2000, and had no previous contact with secondary mental health services for psychosis. Exclusion criteria were evidence of organic psychosis, severe learning difficulties, and transient psychotic symptoms resulting from acute intoxication. Controls with no history of psychosis were randomly selected from the same geographical areas as the cases, and were also between the ages of 16–64 years. To exclude controls who may have experienced undiagnosed psychotic symptoms, all controls were screened using the Psychosis Screening Questionnaire (PSQ) [18].

The study was approved by the local research ethical committee at each study centre. Full details of the study methods have been described previously [19].

### Data collection

The Childhood Experience of Care and Abuse Questionnaire (CECA.Q) [20] was used to elicit information on experiences of childhood adversity before the age of 16. Specifically, we included in these analyses data on physical abuse from the main parent figures and sexual abuse by any person at least 5 years older than the victim. Screening questions relating to physical and sexual abuse were read out to all participants, and positive responses were followed up with more detailed questions. Researchers then used published guidelines to score the severity of the responses in a standardised manner [20]. The measure has satisfactory levels of concurrent validity and test–retest reliability within clinical and non-clinical populations [20, 21] as well as within patients with psychosis [22]. For data analysis, responses from the CECA.Q were dichotomised into severe, and no or non-severe experiences of sexual and physical abuse separately, using the most conservative published cut-points ( [20]; see [5] for further details). The group with none or non-severe instances of sexual and physical abuse is referred to as the ‘no abuse’ group within the categorical analyses presented in this paper.

The Significant Others Scale (SOS) [23] is a self-report questionnaire which measures perceived and ideal levels of practical and emotional support on a seven-point scale from up to seven significant people (e.g. father, partner). The SOS also elicits the number of significant others in participants’ social networks, as well as a discrepancy score (a measure of satisfaction) between ideal and perceived levels of emotional and practical support separately. The measure has been shown to have satisfactory concurrent and construct validity and test–retest reliability [24].

Participants completed the Medical Research Council (MRC) Socio-demographic Schedule [25] to collect data on age, gender, current employment status, education level, parental social class at participant’s birth, and ethnicity. This schedule was also used to collect data on the individual’s current living circumstances, current and long-term relationship status, number of confidants, and frequency of contact with friends and family. Finally, we used the Family Interview for Genetic Studies (FIGS) [26] to obtain data on history of mental health problems in parents (specifically psychosis, mania and depression).

### Data analysis

Logistic regression was used to obtain the crude and adjusted odds ratios of the relationships between case–control status and both physical abuse and sexual abuse. This was done first with the sample unstratified, and then stratified by gender, to investigate the effects separately in men and women, with interaction terms fitted and likelihood ratio tests conducted to test the difference in odds ratios between men and women.

To assess whether social support modified the association between childhood adversity and psychosis, interaction terms were fitted to logistic models and likelihood ratio tests conducted to determine whether the interaction terms improved model fit. Subsequently, the same analyses were repeated, stratified by gender.

To test our hypothesis that there would be differences between men and women in the associations between childhood abuse, social support in adulthood, and psychosis (i.e. a three-way interaction), we first created a four-level ordinal variable to represent all combinations of presence and absence of abuse and social support (i.e. 0—no abuse and high support; 1—no abuse and low support; 2—abuse and high support; 3—abuse and low support). Interaction terms were then fitted to the model and likelihood ratio tests conducted to determine whether the interaction terms improved model fit. A liberal *p* value of 0.10 was used for the interaction tests to ensure potentially important interactions were not discarded.

For all adjusted models, potential confounders included were sex (except in analyses stratified by gender), age (16–35 or 36–64 years), ethnicity (white British or other), study centre (London or Nottingham), education (no qualifications or any qualifications), current employment status [unemployed, economically inactive (i.e. students, house-persons), or employed], and any mental illness in parents. All analyses were carried out using STATA version 11 for Windows.

## Results

Of the 390 cases and 391 controls recruited into the AESOP study, 202 cases and 266 controls completed the SOS and were included in these analyses. There was no strong evidence that this subsample of control participants differed from those on whom SOS data were not available in relation to sex (*χ*^2^ = 1.00, *p* = 0.318) or age (*χ*^2^ = 2.32, *p* = 0.128), though control completers were more likely to be of white British origin (*χ*^2^ = 40.32, *p* < 0.001). Compared with cases who did not complete the SOS, cases who completed the questionnaire were more likely to be women (*χ*^2^ = 5.99, *p* = 0.014) and of White British origin (*χ*^2^ = 9.23, *p* = 0.002). There were no differences in age between cases who completed and did not complete the SOS (*χ*^2^ = 1.35, *p* = 0.246).

The basic demographic characteristics of those included in these analyses by case–control status are shown in Table 1. Cases were more often men, were younger, were less likely to be of white British ethnicity, were more likely to have a lower level of education and be currently unemployed, and were more likely to have parents with a mental illness. Moreover, more cases were recruited from the London site. These variables were controlled for in the following analyses.

**Table 1:** Basic demographic characteristics of psychosis cases and controls

	Cases (*N* = 202) *n* (%)	Controls (*N* = 266) *n* (%)	*X* ^2^	df	*p*
Sex			**4.693**	**1**	**0.030**
Male	100 (49.5)	105 (39.5)			
Female	102 (50.5)	161 (60.5)			
Ethnicity			**20.748**	**1**	**<0.001**
White British	107 (53.0)	195 (73.3)			
Other	95 (47.0)	71 (26.7)			
Study centre			**6.630**	**1**	**0.010**
London	96 (47.5)	95 (35.7)			
Nottingham	106 (52.5)	171 (64.3)			
Age			**26.107**	**1**	**<0.001**
16–35	144 (71.3)	127 (47.7)			
36–65	58 (28.7)	139 (52.3)			
Parental mental illness^a^			**15.179**	**1**	**<0.001**
No	120 (79.5)	220 (92.8)			
Yes	31 (20.5)	17 (7.2)			
Current employment			**43.542**	**2**	**<0.001**
Employed	66 (32.7)	145 (54.5)			
Economically inactive	38 (18.8)	68 (25.6)			
Unemployed	98 (48.5)	53 (19.9)			
Highest education^b^			**5.477**	**1**	**0.019**
Any qualifications	142 (70.7)	212 (80.0)			
School: no qualifications	59 (29.3)	53 (20.0)			
Parental social class^c^			0.615	2	0.735
Managerial, professional	39 (27.1)	60 (26.1)			
Intermediate	38 (26.4)	54 (23.5)			
Routine/manual	67 (46.5)	116 (50.4)			

Bold values indicate statistically significant results

*df* degrees of freedom

^a^Data missing from 51 cases and 29 controls. Mental illness includes psychosis, depression and mania

^b^Data missing from 1 case and 1 control

^c^Data missing from 58 cases and 36 controls

Table 2 presents comparisons between cases and controls on variables indicative of social isolation and levels of perceived support in adulthood. Cases, compared with controls, were less likely to live with others (other than family), and less likely to have a history of being in a long-term relationship. Cases also had less frequent contact with friends, had fewer confidants, fewer significant others, and were more likely to have a social network comprising only of family members. In addition, cases perceived themselves as having less emotional and practical support.

**Table 2:** Comparison of social variables between psychosis cases and controls

	Cases (*N* = 202) *n* (%)	Controls (*N* = 266) *n* (%)	*χ* ^2^	df	*p*
Current living circumstances			**61.547**	**2**	**<0.001**
Others	58 (29.0)	161 (61.2)			
Relatives	64 (32.0)	22 (8.4)			
Alone	78 (39.0)	80 (30.4)			
Current relationship status			**37.167**	**1**	**<0.001**
In a relationship	72 (35.8)	171 (64.3)			
Single	129 (64.2)	95 (35.7)			
Long-term relationship status			**55.630**	**1**	**<0.001**
In a relationship	89 (44.3)	205 (78.0)			
Single	112 (55.7)	58 (22.0)			
Current contact with friends			**19.526**	**2**	**<0.001**
Daily	73 (37.2)	149 (57.5)			
Weekly	71 (36.2)	71 (27.4)			
Less that weekly	52 (26.6)	39 (15.1)			
Current contact with family			3.413	2	0.181
Daily	92 (47.2)	98 (38.9)			
Weekly	66 (33.8)	104 (41.3)			
Less that weekly	37 (19.0)	50 (19.8)			
Presence of close confidants			**42.648**	**1**	**<0.001**
Yes	142 (72.5)	251 (94.4)			
No	54 (27.5)	15 (5.6)			
Current number of significant others as identified by the SOS			**17.754**	**2**	**<0.001**
0–2	51 (25.3)	28 (10.5)			
3–5	83 (41.1)	129 (48.5)			
6–7	68 (33.6)	109 (41.0)			
Current number of individuals whose significant others are family only			**17.813**	**1**	**<0.001**
Yes	79 (39.1)	56 (21.2)			
No	123 (60.9)	208 (78.8)			

			*t*	df	*p*
Average perceived emotional support					
Mean (sd)	10.61 (2.57)	11.48 (1.93)	**4.14**	**464**	**<0.001**
Average perceived practical support					
Mean (sd)	9.92 (2.36)	10.83 (2.02)	**4.49**	**464**	**<0.001**

Bold values indicate statistically significant results

*df* degrees of freedom, *sd* standard deviation, *SOS* Significant Others Scale

### Childhood adversity, gender and psychosis

Cases included in the analyses were around two times more likely to report severe childhood physical abuse compared with controls (OR 2.05, 95 % CI 1.22–3.46, *p* = 0.006), and this association held after adjusting for age, ethnicity, study centre, parental history of mental illness, employment status and education level (adj. OR 1.89, 95 % CI 1.05–3.39, *p* = 0.034). When stratified by gender, female psychosis cases were approximately three times more likely to report severe physical abuse before 16 years of age compared with women in the control group (OR 3.31, 95 % CI 1.56–7.03, *p* < 0.001), though there was no evidence of an association between physical abuse and psychosis in men (OR 1.22, 95 % CI 0.58–2.54, *p* = 0.597). A likelihood ratio test of the difference in odds ratios revealed an interaction between gender and physical abuse (*χ*^2^ = 3.55, *p* = 0.059). This was only slightly attenuated following adjustment for potential confounders (*χ*^2^ = 3.16, *p* = 0.076).

In contrast, reports of severe childhood sexual abuse were only marginally more likely in cases compared with controls (OR 1.44, 95 % CI 0.82–2.53, *p* = 0.199) and this did not reach conventional levels of statistical significance. However, when we repeated these analyses stratifying by gender, the results indicated that whilst there was no association between childhood sexual abuse and psychosis in men (OR 0.76, 95 % CI 0.26–2.24, *p* = 0.613), women in the cases group were twice as likely to report experience of severe sexual abuse before the age of 16 than female controls (OR 2.21, 95 % CI 1.11–4.41, *p* = 0.021). A likelihood ratio test of the difference in odds ratios between men and women showed an interaction with sexual abuse (*χ*^2^ = 2.78, *p* = 0.096), which remained robust following adjustment for potential confounders (*χ*^2^ = 2.90, *p* = 0.089).

### Childhood adversity, social support and psychosis

There was evidence that the impact of severe physical abuse in childhood on odds of psychosis varied by number of significant others in adulthood (see Table 3). The adjusted odds ratio for those who reported abuse and had 5 or more significant others in their social networks was 0.99 (95 % CI 0.42–2.36), compared with 3.24 (95 % CI 1.42–7.38) for those who reported abuse and had fewer than 5 significant others (χ^2^ = 3.90, *p* = 0.048). More tentatively, the impact of physical abuse on odds of psychosis varied by perceived practical support, in that the odds ratio for those who reported physical abuse and high practical support was 1.18 (95 % CI 0.45–3.08), compared with 2.43 (95 % CI 1.11–5.33) for those who reported abuse and did not receive practical support. However, the magnitude of this variation did not reach conventional levels of statistical significance (*χ*^2^ = 1.35, *p* = 0.245). In contrast, there was no evidence that the impact of physical abuse varied by current perceived emotional support.

**Table 3:** Severe childhood abuse and current social support by case–control status

			Unadjusted		Adjusted^a^	
			OR (95 % CI)	*p*	OR (95 % CI)	*p*
Severe physical abuse						
Perceived emotional support	Low support	No abuse	1		1	
		Abuse	**1.96 (1.01–3.79)**	**0.043**	1.74 (0.83–3.67)	0.145
	High support	No abuse	1		1	
		Abuse	1.91 (0.80–4.56)	0.136	2.16 (0.83–5.59)	0.112
			LR test: *χ* ^2^ = 0.00, *p* = 0.969		LR test: *χ* ^2^ = 0.12, *p* = 0.726	
Perceived practical support	Low support	No abuse	1		1	
		Abuse	**2.59 (1.28–5.24)**	**0.006**	**2.43 (1.11–5.33)**	**0.027**
	High support	No abuse	1		1	
		Abuse	1.06 (0.43–2.60)	0.906	1.18 (0.45–3.08)	0.736
			LR test: *χ* ^2^ = 2.42, *p* = 0.120		LR test: *χ* ^2^ = 1.35, *p* = 0.245	
Number of significant others	0–4 others	No abuse	1		1	
		Abuse	**3.39 (1.59–7.25)**	**<0.001**	**3.24 (1.42–7.38)**	**0.005**
	5–7 others	No abuse	1		1	
		Abuse	1.13 (0.52–2.43)	0.761	0.99 (0.42–2.36)	0.985
			**LR test: χ** ^**2**^ **=** **4.11,** ***p*** **=** **0.043**		**LR test:** ***χ*** ^**2**^ **=** **3.90,** ***p*** **=** **0.048**	
Severe sexual abuse						
Perceived emotional support	Low support	No abuse	1		1	
		Abuse	**2.29 (1.06–4.95)**	**0.031**	2.12 (0.86–5.20)	0.102
	High support	No abuse	1		1	
		Abuse	0.74 (0.29–1.89)	0.523	0.93 (0.32–2.70)	0.899
			**LR test:** ***χ*** ^**2**^ **=** **3.41,** ***p*** **=** **0.065**		LR test: *χ* ^2^ = 1.37, *p* = 0.242	
Perceived practical support	Low support	No abuse	1		1	
		Abuse	1.30 (0.62–2.74)	0.492	1.10 (0.47–2.62)	0.822
	High support	No abuse	1		1	
		Abuse	1.57 (0.63–3.90)	0.324	2.03 (0.71–5.78)	0.184
			LR test: *χ* ^2^ = 0.10, *p* = 0.748		LR test: *χ* ^2^ = 0.78, *p* = 0.377	
Number of significant others	0–4 others	No abuse	1		1	
		Abuse	1.96 (0.88–4.38)	0.094	1.90 (0.73–4.99)	0.191
	5–7 others	No abuse	1		1	
		Abuse	1.03 (0.45–2.32)	0.951	1.12 (0.44–2.80)	0.816
			LR test: *χ* ^2^ = 1.24, *p* = 0.266		LR test: *χ* ^2^ = 0.63, *p* = 0.427	

Bold values indicate statistically significant results

*OR* odds ratio, *CI* confidence interval, *LR* likelihood ratio

^a^Adjusted for gender, age, ethnicity, education, current employment, parental history of mental illness, and study centre

In addition, and again tentatively, the impact of severe sexual abuse on odds of psychosis varied by perceived emotional support, whereby the odds ratio for those who reported sexual abuse and high emotional support was 0.93 (95 % CI 0.32–2.70), compared with 2.12 (95 % CI 0.86–5.20) for those who reported abuse and did not perceive themselves as having sufficient emotional support, though the magnitude of this variation did not reach conventional levels of statistical significance (*χ*^2^ = 1.37, *p* = 0.242). In contrast, there was no evidence that the impact of sexual abuse varied by current levels of perceived practical support or number of significant others.

### Gender differences in the association between childhood adversity, social support and psychosis

When stratified by gender, there were no clear associations between childhood physical or sexual abuse, current social support and odds of psychosis in men (Table 4). In contrast, women were around four times more likely to be a case following childhood physical abuse compared with those who reported no exposure to abuse if they currently did not perceive themselves as having sufficient emotional support (OR 4.04, 95 % CI 1.47–11.09) or sufficient practical support (OR 4.90, 95 % CI 1.65–14.57), and were over six times more likely to be a case if they had fewer than five significant others (OR 6.14, 95 % CI 1.80–21.00). When adjusted for confounders, individuals exposed to abuse and who reported low perceived practical support and fewer significant others still had greater odds of psychosis compared with those who had experienced no abuse (adj. OR 4.33, 95 % CI 1.39–13.43 and adj. OR 6.73, 95 % CI 1.96–23.12, respectively). Moreover, a likelihood ratio test for the difference in odds ratios between those who had few significant others compared with those with five or more others demonstrated an interaction with physical abuse in women (*χ*^2^ = 3.55, *p* = 0.060). In contrast, there was no variation in odds of psychosis in women exposed to physical abuse according to levels of perceived emotional support in adulthood after adjusting for confounders (low emotional support: adj. OR 2.87, 95 % CI 0.98–8.37; high emotional support: adj. OR 3.56, 95 % CI 1.22–17.33).

**Table 4:** Prevalence of severe childhood abuse and current social support for psychosis cases and controls by gender

			Males				Females			
			Unadjusted		Adjusted^a^		Unadjusted		Adjusted^a^	
			OR (95 % CI)	*p*	OR (95 % CI)	*p*	OR (95 % CI)	*p*	OR (95 % CI)	*p*
Severe physical abuse										
Perceived emotional support	No support	No abuse	1		1		1		1	
		Abuse	1.00 (0.40–2.51)	1.000	1.20 (0.39–3.72)	0.749	**4.04 (1.47–11.09)**	**0.003**	2.87 (0.98–8.37)	0.054
	Support	No abuse	1		1		1		1	
		Abuse	1.54 (0.44–5.35)	0.495	1.08 (0.24–4.84)	0.919	2.22 (0.65–7.52)	0.189	**3.56 (1.00–12.70)**	**0.050**
			LR test: *χ* ^2^ = 0.30, *p* = 0.585		LR test: *χ* ^2^ = 0.01, *p* = 0.910		LR test: *χ* ^2^ = 0.56, *p* = 0.456		LR test: *χ* ^2^ = 0.07, *p* = 0.799	
Perceived practical support	No support	No abuse	1		1		1		1	
		Abuse	1.40 (0.54–3.65)	0.489	1.51 (0.45–5.09)	0.504	**4.90 (1.65–14.57)**	**0.002**	**4.33 (1.39–13.43)**	**0.011**
	Support	No abuse	1		1		1		1	
		Abuse	0.78 (0.21–2.82)	0.702	0.76 (0.18–3.29)	0.716	1.33 (0.37–4.76)	0.657	1.59 (0.42–6.12)	0.497
			LR test: *χ* ^2^ = 0.52, *p* = 0.471		LR test: *χ* ^2^ = 0.51, *p* = 0.476		LR test: *χ* ^2^ = 2.44, *p* = 0.118		LR test: *χ* ^2^ = 1.29, *p* = 0.255	
Number of significant others	0–4 others	No abuse	1		1		**1**		**1**	
		Abuse	2.07 (0.76–5.62)	0.145	1.44 (0.44–4.71)	0.551	**6.14 (1.80–21.00)**	**<0.001**	**6.73 (1.96–23.12)**	**0.002**
	5–7 others	No abuse	1		1		1		1	
		Abuse	0.63 (0.20–1.98)	0.424	0.77 (0.19–3.18)	0.722	1.68 (0.58–4.82)	0.333	1.37 (0.42–4.47)	0.600
			LR test: *χ* ^2^ = 2.43, *p* = 0.119		LR test: *χ* ^2^ = 0.44, *p* = 0.506		LR test: *χ* ^2^ = 2.59, *p* = 0.107		**LR test:** ***χ*** ^**2**^ **=** **3.55,** ***p*** **=** **0.060**	
Severe sexual abuse										
Perceived emotional support	No support	No abuse	1		1		1		1	
		Abuse	0.98 (0.23–4.19)	0.975	0.71 (0.09–5.61)	0.741	**4.41 (1.60–12.16)**	**0.002**	**3.64 (1.25–10.61)**	**0.018**
	Support	No abuse	1		1		1		1	
		Abuse	0.55 (0.10–3.06)	0.485	0.23 (0.03–1.98)	0.180	0.90 (0.29–2.81)	0.849	1.25 (0.37–4.19)	0.721
			LR test: *χ* ^2^ = 0.26, *p* = 0.612		LR test: *χ* ^2^ = 0.58, *p* = 0.447		**LR test:** ***χ*** ^**2**^ **=** **4.43,** ***p*** **=** **0.035**		LR test: *χ* ^2^ = 1.71, *p* = 0.191	
Perceived practical support	No support	No abuse	1		1		1		1	
		Abuse	0.65 (0.16–2.61)	0.541	0.37 (0.06–2.32)	0.289	2.00 (0.79–5.09)	0.138	1.52 (0.55–4.18)	0.422
	Support	No abuse	1		1		1		1	
		Abuse	0.87 (0.15–5.11)	0.873	0.53 (0.05–5.26)	0.590	2.33 (0.78–7.01)	0.120	3.14 (0.94–10.48)	0.063
			LR test: *χ* ^2^ = 0.06, *p* = 0.804		LR test: *χ* ^2^ = 0.06, *p* = 0.805		LR test: *χ* ^2^ = 0.04, *p* = 0.834		LR test: *χ* ^2^ = 0.81, *p* = 0.369	
Number of significant others	0–4 others	No abuse	1		1		1		1	
		Abuse	0.88 (0.23–3.37)	0.847	0.59 (0.09–3.92)	0.584	**3.31 (1.11–9.87)**	**0.023**	**3.55 (1.07–11.77)**	**0.038**
	5–7 others	No abuse	1		1		1		1	
		Abuse	0.65 (0.10–4.16)	0.647	0.26 (0.03–2.13)	0.209	1.51 (0.59–3.89)	0.386	1.49 (0.55–4.07)	0.437
			LR test: *χ* ^2^ = 0.07, *p* = 0.798		LR test: *χ* ^2^ = 0.34, *p* = 0.562		LR test: *χ* ^2^ = 1.15, *p* = 0.284		LR test: *χ* ^2^ = 1.20, *p* = 0.273	

Bold values indicate statistically significant results

*OR* odds ratio, *CI* confidence interval, *LR* likelihood ratio

^a^Adjusted for age, ethnicity, education, current employment, parental history of mental illness, and study centre

Similar associations, although weaker, were found in relation to childhood sexual abuse. Women who had experienced sexual abuse as a child and who currently had low perceived emotional support were approximately four times more likely to be a case (OR 4.41, 95 % CI 1.60–12.16) compared with those who experienced no abuse. This association held after adjusting for potential confounders (adj. OR 3.64, 95 % CI 1.25–10.61). A likelihood ratio test of the difference in odds ratios between those who had little emotional support compared with those with high support did not reach significance (*χ*^2^ = 1.71, *p* = 0.191). Moreover, women who had experienced sexual abuse as a child and who currently had few significant others were around three times more likely to be a case (OR 3.31, 95 % CI 1.11–9.87) compared with those who had experienced no abuse, and this association held after adjusting for potential confounders (adj. OR 3.55, 95 % CI 1.07–11.77). Lastly, women with a history of sexual abuse who perceived themselves as having *high* practical support were three times more likely to be a case compared with those who reported no abuse (adj. OR 3.14, 95 % CI 0.94–10.48). Formal tests for three-way interaction by gender, however, did not reveal any significant differences between men and women in the modifying effects of social support between childhood adversity and adult psychosis (see Online Resource 1).

## Discussion

Our data are suggestive of potentially important relationships between psychosis and perceived social support and social network variables in those who report experience of early adversity, particularly in those with a history of physical abuse. Firstly, there are clear differences between cases and controls in levels of support at time of interview; it is apparent that psychosis cases were more socially isolated, and perceived themselves as having less support. In the unstratified sample, we found that the number of significant others modified the effect of childhood physical abuse on odds of psychosis, whereby those who had reported physical abuse had lower odds of psychosis if they had a larger number of significant others at the time of interview. This was independent of a range of potential confounders. We found no other evidence of interactions between childhood physical abuse and other forms of social support, nor between childhood sexual abuse and any form of social support.

When the sample was stratified by gender, there was evidence of variation in the associations within men and women. Notably, women who had reported physical abuse as a child and had a larger number of significant others in adulthood were less likely to be diagnosed with psychosis than those with fewer significant others. Nevertheless, there was no strong evidence in favour of our hypothesis that there would be significant differences between men and women in the associations between reported abuse, social support, and psychosis. However, findings of continued high odds in women who reported physical abuse or sexual abuse, and who reported high emotional support and high practical support, respectively, may tentatively suggest that particular dimensions of social support may have differential effects on development of psychosis following different forms of maltreatment. Clearly though, what is apparent from the findings is that a lack of support in adulthood was associated with substantially increased odds of psychosis in women who reported childhood physical abuse.

### Methodological limitations

There are several limitations which should be considered when interpreting the findings of this study. Firstly, we relied on retrospective reports of abuse, which have been criticised due to the potential of recall bias especially in those with severe mental illness [27]. However, reports of childhood abuse by individuals with psychosis have been shown to be stable over a seven-year time-period and independent of current symptoms [22], and prospective and retrospective reports of maltreatment have been equally associated with an elevated risk of psychopathology [28]. It is also important to bear in mind that longitudinal studies are not always feasible or justified, due to the large number of participants needed to generate enough cases of psychosis, and that questioning children about recent adverse events may cause substantial distress.

Secondly, cases with psychosis were not asked retrospectively about indices of social support, and therefore the data reflect size of social network and levels of support at the time of interview. Whilst it is possible that perceived social support and social network variables in individuals with psychosis may be consequentially influenced by symptomatology (e.g. those experiencing persecutory delusions or depressive symptoms may undervalue their social relationships) or contact with mental health services, there is limited evidence to suggest that such a “social network crisis” does exist directly following the onset of psychosis [29–31]. Nevertheless, future research would benefit from investigating the resilience effects of social support earlier on in the developmental trajectory in those with and without a history of childhood adversity.

In a similar vein, the data collected was cross-sectional in nature, and thus causation cannot necessarily be implied. Nonetheless, evidence from a recent systematic review [16] and from an increasing body of more recently published research (e.g. [32–35]) are suggestive of poor support and deficits in network functioning preceding overt psychotic symptoms. General population studies are a valuable tool for investigating the extended psychosis phenotype; a well-designed longitudinal study conducted in a sample of general population adolescents over 3 years tested the bidirectional association between social functioning and separate dimensions of subclinical positive psychotic experiences [36]. The authors reported a unidirectional relationship such that poor social functioning predicted the development of bizarre experiences and persecutory ideation over time, but not vice versa, thus raising the possibility that a lack of support is indeed contributory to the formation of subclinical psychotic experiences as well as to clinical disorder. There is, however, an evident need to explore antecedents to social isolation in individuals at risk of psychosis—amongst other factors, it could be a consequence of alterations in neurodevelopment in those at psychometric risk, or it could be partially attributable to stressful and traumatic events in childhood or adulthood which could lead to deterioration in social support.

Finally, whilst we carried out a formal test of the differences in the protective effects of social support between men and women (Online Resource 1), this did not result in any statistically significant findings. Nevertheless, the effects are such that they merit reporting given that the results are in the hypothesised direction and that the sample size is such that it would be difficult to detect a significant effect. However, we acknowledge that caution is warranted when interpreting the results, and future research would benefit from the use of larger samples in order to allow firmer conclusions to be drawn.

### Mechanisms of social support and future research

Previous research has shown that women benefit more from the buffering effects of social support than men, in particular in reducing the risk of experiencing symptoms of depression [15, 37], yet the reason why remains unclear. This current study provided tentative support for such a gender difference in the protective effects of social support following childhood maltreatment in psychosis. It is evident that women benefit more from social support than men, yet the reason why remains very unclear. There are several stages within the support process in which individuals may face barriers to receiving the full positive impact of social support [38]; these include recognising a need for help, deciding what aspect of support may be most beneficial in the situation, and whether there are others who can and are willing to provide the support they need. Then support must be asked for, or if it is freely offered, then the individual has to decide whether to accept it. It is possible that men and women differ in one or more of these stages thus leading to findings of differential health benefits of social support between genders.

Understanding the differences between genders in the positive effects of social support is also impeded by a lack of understanding of how support buffers stress. Several potential mechanisms have been proposed. Firstly, tangible aid or information may change the nature of stressful events [39]. However, research has shown that simply perceiving that one has more available support predicts better adjustment to stress [8, 38], which may act by altering the appraisal of the stressful event [40]. In addition, social support may alter the individual’s self-esteem or their perceived control over their environment, which have both been directly associated with a better adaptation to stressful events [41, 42]. Men and women have been found to differ in the perceived stressfulness of events and their mastery over stressful events [43], and thus men may be less likely to seek support from others close to them.

It has also been proposed that actively seeking help or receiving help results in greater ego-costs for men than women [44] due to traditional gender roles which reinforce independence and low emotional disclosure in men [45]. Divulging the need for help in men, more so than women, may also precipitate another potential cost of decreased perception of self-efficacy or control. Finally, having a larger social network may change the number of resources available to address the stressful event, or increase the likelihood of normative health behaviours [46] or access to normalising explanations for anomalous experiences and abnormal beliefs [47]. This last theory may explain the findings found in this study population, which demonstrated that size of social network was most strongly associated with resilience to psychosis following abuse, especially in women.

These mechanisms are all of potential importance in understanding the apparent relationship between childhood adversity and risk of psychosis. At a critical developmental period, childhood physical and sexual abuse can create a lasting cognitive vulnerability in the form of negative schematic beliefs, resulting in low self-esteem, which in adulthood could lead to an inability to deal with stress and increase vulnerability to psychosis [48]. In addition, and in line with the stress-vulnerability model of psychosis, previous exposure to child abuse may lead to stress sensitisation whereby the individual is found to be more sensitive and perceive higher levels of stress following adverse events [49]. In short, initial research suggests that low self-esteem and negative schemata, and stress sensitisation contribute to the development of psychosis, and may be a long-term consequence of having experienced adverse childhood events. Given that social support in adulthood has been shown to modify each of these variables to promote emotional well-being and to decelerate decompensation to other negative mental health outcomes, it is apparent that exploring the protective effects of social support in the relationship between childhood adversity and psychosis, and the mediating mechanisms, is an important and valuable next step.

Lastly, it would be interesting to investigate further the effects of levels of support and size of networks that exist around the time of abuse, as well as different sources of support, in mitigating the effects of childhood adversity on development of psychosis. As most instances of childhood maltreatment (except sexual abuse) are likely to occur within the family, the individual may have a more negative perception of family support, than perception of friend support, and thus the former may not have a substantial effect in protecting against the development of psychosis. Indeed, in female adult victims of child abuse, perceived friend support, but not family support, has been found to act as a buffer against PTSD [9] and symptoms of depression in adulthood [37]. Differential effects of sources of support, and of support closer to the time of adversity, have yet to be explored in psychosis.

## Conclusion

Tentative evidence of gender differences was found in this study whereby size of social network appeared to have a buffering effect against adult psychosis only in women who experienced severe abuse in childhood. Indeed, psychosis in women has been postulated to be a more socially reactive condition [50]. Particularly for women who have experienced child maltreatment, these findings highlight the potential importance of social support interventions that strengthen social network systems and perceptions of social support in order to provide resilience against developing psychosis. Further research utilising larger sample sizes and prospectively collected data from childhood through to adulthood is required to fully ascertain the role of social support in increasing resilience to psychosis following exposure to childhood maltreatment.

## Electronic supplementary material

Supplementary material 1 (DOCX 23 kb)
