# Supplementary material for: Gender differences in the association between childhood physical and sexual abuse, social support and psychosis
Source: Soc Psychiatry Psychiatr Epidemiol. 2015 Apr 18;50(10):1489–500. doi: 10.1007/s00127-015-1058-6 (PMC4589555; doi:10.1007/s00127-015-1058-6)
Supplement: Supplementary file 1 — Supplementary material 1 (DOCX 23 kb) [file 127_2015_1058_MOESM1_ESM.docx]

**Social Psychiatry and Psychiatric Epidemiology**

**Gender Differences in the Association between Childhood Physical and Sexual Abuse, Social Support and Psychosis**

Charlotte Gayer-Anderson PhD*^1^, Helen L Fisher PhD^2^, Paul Fearon MRCPsych^3^, Gerard Hutchinson MRCPsych^4^, Kevin Morgan PhD^5^, Paola Dazzan MRCPsych^6^, Jane Boydell MRCPsych^6^, Gillian A Doody FRCPsych^7^, Peter B Jones FRCPsych^8^, Robin M Murray FRCPsych^6^, Thomas K Craig FRCPsych^1,6^ and Craig Morgan PhD^1^

**Correspondence to:** Charlotte Gayer-Anderson, Section of Social Psychiatry, Health Service and Population Research Department, Institute of Psychiatry, Psychology & Neuroscience, King’s College London, 16 De Crespigny Park, London SE5 8AF, UK. Tel: 020 7848 5074. E-mail: [charlotte.gayer-anderson@kcl.ac.uk](mailto:charlotte.gayer-anderson@kcl.ac.uk)

^1^NIHR Biomedical Research Centre, and Section of Social Psychiatry, Health Service and Population Research Department, Institute of Psychiatry, Psychology & Neuroscience, King’s College London, UK; ^2^MRC Social, Genetic and Developmental Psychiatry Centre, Institute of Psychiatry, Psychology & Neuroscience, King’s College London, UK; ^3^Department of Psychiatry, Trinity College Dublin, St Patrick's University Hospital, Ireland; ^4^Psychiatry Unit, University of the West Indies, Trinidad; ^5^Department of Psychology, University of Westminster, London, UK; ^6^NIHR Biomedical Research Centre, and Psychosis Studies Department, Institute of Psychiatry, Psychology & Neuroscience, King’s College, London, UK; ^7^Division of Psychiatry and Applied Psychology, University of Nottingham, Nottingham, UK; ^8^Department of Psychiatry, University of Cambridge, Cambridge, UK

**Electronic Online Resource 1.** Interaction between severe childhood abuse and current social support for psychosis cases and controls by gender

|  | | **UNADJUSTED** | | | | | **ADJUSTED *** | | | |
| --- | --- | --- | --- | --- | --- | --- | --- | --- | --- | --- |
| **SEVERE PHYSICAL ABUSE** | | **Males** | | **Females** | | | **Males** | | **Females** | |
|  |  | **OR (95% CI)** | ***p*** | **OR (95% CI)** | ***p*** | | **OR (95% CI)** | ***p*** | **OR (95% CI)** | ***p*** |
| **PERCEIVED EMOTIONAL SUPPORT** | **No abuse and high support** | 1 |  | 1 |  | | 1 |  | 1 |  |
|  | **No abuse and low support** | 1.54 (0.79-3.00) | 0.206 | 0.98 (0.51-1.87) | 0.942 | | 1.06 (0.49-2.32) | 0.881 | 0.92 (0.44-1.91) | 0.819 |
|  | **Abuse and high support** | 1.54 (0.45-5.29) | 0.494 | 2.22 (0.66-7.41) | 0.195 | | 1.09 (0.28-4.23) | 0.906 | **4.09 (1.13-14.81)** | **0.032** |
|  | **Abuse and low support** | 1.54 (0.60-3.94) | 0.369 | **3.94 (1.58-9.87)** | **0.003** | | 1.19 (0.41-3.44) | 0.747 | 2.57 (0.89-7.38) | 0.080 |
|  |  |  | | LR test: χ^2^=4.61, p=0.203 | | |  | | LR test: χ^2^=3.41, p=0.333 | |
| **PERCEIVED PRACTICAL SUPPORT** | **No abuse and high support** | 1 |  |  |  | | 1 |  | 1 |  |
|  | **No abuse and low support** | 1.94 (0.99-3.82) | 0.053 | 1.84 (0.96-3.51) | 0.066 | | 1.29 (0.59-2.84) | 0.522 | 1.89 (0.92-3.90) | 0.085 |
|  | **Abuse and high support** | 0.78 (0.22-2.80) | 0.700 | 1.33 (0.38-4.72) | 0.656 | | 0.75 (0.19-2.97) | 0.686 | 1.78 (0.46-6.85) | 0.402 |
|  | **Abuse and low support** | **2.72 (1.03-7.22)** | **0.044** | **8.90 (3.20-25.29)** | **<0.001** | | 1.87 (0.61-5.69) | 0.271 | **8.15 (2.55-26.04)** | **<0.001** |
|  |  |  | | LR test: χ^2^=3.56, p=0.313 | | |  | | LR test: χ^2^=3.61, p=0.306 | |
| **NUMBER OF SIGNIFICANT**  **OTHERS** | **No abuse and 5-7 sig others** | 1 |  | 1 |  | | 1 |  | 1 |  |
|  | **No abuse and 0-4 sig others** | 0.61 (0.31-1.18) | 0.143 | 1.17 (0.60-2.26) | 0.643 | | 0.81 (0.37-1.77) | 0.595 | 1.02 (0.48-2.15) | 0.967 |
|  | **Abuse and 5-7 sig others** | 0.63 (0.20-1.96) | 0.424 | 1.68 (0.59-4.78) | 0.334 | | 0.68 (0.19-2.40) | 0.550 | 1.35 (0.41-4.47) | 0.623 |
|  | **Abuse and 0-4 sig others** | 1.26 (0.47-3.37) | 0.649 | **7.18 (2.41-21.37)** | **<0.001** | | 1.33 (0.44-3.99) | 0.610 | **7.51 (2.23-25.27)** | **0.001** |
|  |  |  | | **LR test: χ^2^=6.59, p=0.086** | | |  | | LR test: χ^2^=4.71, p=0.194 | |
| **SEVERE SEXUAL ABUSE** | | **Unadjusted** | | **Adjusted ^a^** | | | **Unadjusted** | | **Adjusted ^a^** | |
|  |  | **OR (95% CI)** | ***p*** | **OR (95% CI)** | | ***p*** | **OR (95% CI)** | ***p*** | **OR (95% CI)** | ***p*** |
| **PERCEIVED EMOTIONAL SUPPORT** | **No abuse and high support** | 1 |  | 1 |  | | 1 |  | 1 |  |
|  | **No abuse and low support** | 1.40 (0.74-2.63) | 0.298 | 0.84 (0.44-1.63) | 0.615 | | 1.04 (0.50-2.16) | 0.914 | 0.79 (0.38-1.67) | 0.543 |
|  | **Abuse and high support** | 0.55 (0.10-3.01 | 0.488 | 0.90 (0.29-2.79) | 0.849 | | 0.35 (0.05-2.44) | 0.287 | 1.46 (0.43-5.03) | 0.546 |
|  | **Abuse and low support** | 1.37 (0.32-5.91) | 0.676 | **3.72 (1.50-9.24)** | **0.005** | | 0.97 (0.18-5.31) | 0.968 | 2.49 (0.88-7.06) | 0.085 |
|  |  |  | | LR test: χ^2^=3.70, p=0.296 | | |  | | LR test: χ^2^=3.28, p=0.350 | |
| **PERCEIVED PRACTICAL SUPPORT** | **No abuse and high support** | 1 |  | 1 |  | | 1 |  | 1 |  |
|  | **No abuse and low support** | **2.13 (1.12-4.05)** | **0.021** | **2.50 (1.29-4.86)** | **0.007** | | 1.41 (0.67-2.98) | 0.361 | **2.74 (1.31-5.75)** | **0.008** |
|  | **Abuse and high support** | 0.87 (0.15-5.05) | 0.872 | 2.33 (0.79-6.88) | 0.125 | | 0.63 (0.08-4.93) | 0.658 | **3.51 (1.03-12.00)** | **0.045** |
|  | **Abuse and low support** | 1.38 (0.34-5.61) | 0.649 | **5.00 (1.96-12.76)** | **0.001** | | 0.79 (0.16-4.00) | 0.779 | **3.62 (1.26-10.39)** | **0.017** |
|  |  |  | | LR test: χ^2^=2.90, p=0.407 | | |  | | LR test: χ^2^=4.19, p=0.242 | |
| **NUMBER OF SIGNIFICANT**  **OTHERS** | **No abuse and 5-7 sig others** | 1 |  | 1 |  | | 1 |  | 1 |  |
|  | **No abuse and 0-4 sig others** | 0.74 (0.40-1.39) | 0.349 | 1.32 (0.68-2.56) | 0.409 | | 0.95 (0.46-1.96) | 0.897 | 1.30 (0.62-2.72) | 0.494 |
|  | **Abuse and 5-7 sig others** | 0.65 (0.10-4.11) | 0.647 | 1.51 (0.59-3.86) | 0.386 | | 0.42 (0.06-3.07) | 0.394 | 1.54 (0.55-4.34) | 0.412 |
|  | **Abuse and 0-4 sig others** | 0.65 (0.17-2.49) | 0.529 | **4.37 (1.59-12.01)** | **0.004** | | 0.69 (0.14-3.41) | 0.650 | **4.14 (1.26-13.60)** | **0.019** |
|  |  |  | | LR test: χ^2^=5.79, p=0.122 | | |  | | LR test: χ^2^=4.08, p=0.253 | |

OR, odds ratio; CI, confidence interval; LR, likelihood ratio.

^a^ Adjusted for age, ethnicity, education, current employment, parental history of mental illness, and study centre.
